# Obesity and Fibrosis: Setting the Stage for Breast Cancer

**DOI:** 10.3390/cancers15112929

**Published:** 2023-05-26

**Authors:** Genevra Kuziel, Brittney N. Moore, Lisa M. Arendt

**Affiliations:** 1 Cancer Biology Graduate Program, University of Wisconsin-Madison, 1111 Highland Ave, Madison, WI 53705, USA; 2 Department of Comparative Biosciences, University of Wisconsin-Madison, 2015 Linden Drive, Madison, WI 53706, USA

**Keywords:** breast cancer, obesity, mammary gland, cancer-associated fibroblasts, macrophages, adipocytes, extracellular matrix

## Abstract

**Simple Summary:**

Obesity is a significant risk factor for breast cancer in postmenopausal women. Regardless of menopausal status, women with obesity have an elevated incidence of metastasis and a decreased response to treatment. Understanding how obesity promotes the development and progression of breast cancer could improve interventions for women with obesity. Breast tissue contains fat. In response to obesity, breast tissue fat contains more collagen that surrounds the fat cells and epithelial cells. Supportive cells within the fat increase collagen production, which elevates the stiffness of the tissue. Inflammatory cells called macrophages interact with other supportive cells within the fat to increase collagen in the surrounding breast tissue. While weight loss is recommended to reduce obesity, the long-term effects on improving inflammation and the amount of collagen are less clear. Increased collagen and inflammation within breast tissue may enhance the risk for tumor development and promote characteristics associated with a worsened prognosis.

**Abstract:**

Obesity is a rising health concern and is linked to a worsened breast cancer prognosis. Tumor desmoplasia, which is characterized by elevated numbers of cancer-associated fibroblasts and the deposition of fibrillar collagens within the stroma, may contribute to the aggressive clinical behavior of breast cancer in obesity. A major component of the breast is adipose tissue, and fibrotic changes in adipose tissue due to obesity may contribute to breast cancer development and the biology of the resulting tumors. Adipose tissue fibrosis is a consequence of obesity that has multiple sources. Adipocytes and adipose-derived stromal cells secrete extracellular matrix composed of collagen family members and matricellular proteins that are altered by obesity. Adipose tissue also becomes a site of chronic, macrophage-driven inflammation. Macrophages exist as a diverse population within obese adipose tissue and mediate the development of fibrosis through the secretion of growth factors and matricellular proteins and interactions with other stromal cells. While weight loss is recommended to resolve obesity, the long-term effects of weight loss on adipose tissue fibrosis and inflammation within breast tissue are less clear. Increased fibrosis within breast tissue may increase the risk for tumor development as well as promote characteristics associated with tumor aggressiveness.

## 1. Introduction

Obesity has rapidly become a global health epidemic. Current estimates show that worldwide obesity among adults has nearly tripled since 1975 [1,2]. Obesity enhances the risk of multiple types of cancer [3,4,5]. Postmenopausal women with obesity are at a higher risk for a breast cancer diagnosis, particularly hormone receptor-positive breast cancers, which express estrogen receptor alpha (ERα) and progesterone receptor (PR) [6,7]. While still rare, the risk of ERα^+^ breast cancer is also increased with obesity in men [8]. Hormone receptor-positive tumors can further be divided into molecular subtypes Luminal A and Luminal B based on gene expression levels of ERα, and PR, proliferation markers, and human epidermal growth factor receptor 2 (HER2) expression [9]. Luminal B subtype tumors generally have lower expression of ERα and PR than Luminal A subtype tumors, greater proliferation, are of a higher grade, and may be less responsive to endocrine therapies [10,11]. Women with obesity are more likely to develop tumors of the Luminal B subtype [12,13]. While obesity increases breast cancer risk in postmenopausal women, obesity reduces breast cancer risk in the total population of young women [14]. However, obesity enhances other risk factors in premenopausal women, such as a family history of breast cancer or inherited breast cancer 1 or 2 (BRCA1/2) mutations [15,16,17]. Although triple-negative breast cancers, which lack expression of ERα, PR, and HER2, are more frequently diagnosed in premenopausal patients [18], the impact of obesity on the risk of development of triple-negative breast cancer in premenopausal women is less clearly defined [19,20,21], which may be in part due to underlying familial risk factors that impact younger women. 

Regardless of menopausal status, recent meta-analyses involving large numbers of patients have provided evidence that obesity is associated with an increased risk of recurrence of approximately 35–40% in breast cancer patients [22]. When compared with lean women, women with obesity are more likely to be diagnosed with larger, high-grade tumors [23,24,25]. Obesity is also an independent prognostic factor for developing distant metastases due to breast cancer [26], leading to shorter disease-free periods and overall survival rates [22,24,26,27]. Obesity at the time of diagnosis is also associated with decreased response to multiple cancer therapies [28,29].

**Figure 1:**
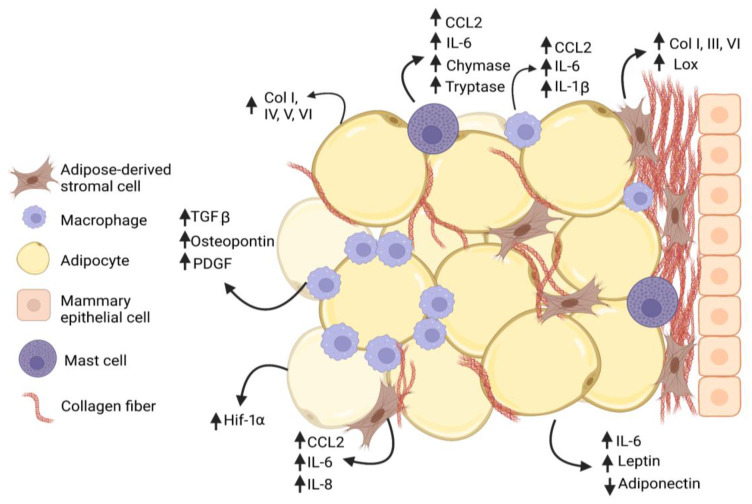
Interactions among cells within mammary adipose tissue enhance fibrosis in obesity. Adipose-derived stromal cells (ASC) and adipocytes increase (↑) secretion of collagen family members as well as growth factors and cytokines that promote adipose tissue fibrosis in obesity. Macrophages and mast cells are recruited into the obese adipose tissue microenvironment and interact with ASC to promote deposition of collagen and other extracellular matrix (ECM) proteins. Macrophages form characteristic crown-like structures surrounding adipocytes. Increased collagen, growth factors, and cytokines are produced by multiple cell types, while adiponectin secretion is decreased (↓) by adipocytes.

Obesity has been correlated with desmoplasia in human breast tumors [30]. Desmoplastic tumors, characterized by increased numbers of cancer associated fibroblasts (CAF) and deposition of fibrillar collagens within the stroma, are associated with diminished survival in breast cancer patients [31,32,33,34]. In addition to collagen, the accumulation of other extracellular matrix (ECM) components, such as hyaluronic acid, is associated with the generation of a more robust stroma, leading to tumor growth and a worsened prognosis [35]. Preclinical models have shown that tumor accumulation of these matrix components is associated with increased tumor interstitial pressure, the collapse of tumor vasculature, and the consequent development of a hypoxic phenotype [36]. Tumor desmoplasia is associated with reduced relapse-free survival following chemotherapy [32,33]. This review will further discuss how obesity enhances fibrosis within the breast stroma (Figure 1) to promote breast cancer risk and tumor aggressiveness.

## 2. Obesity and Adipose Tissue Fibrosis

The percentage volume of subcutaneous adipose tissue in the breast is variable and can comprise 56% of breast weight [36]. ECM proteins in adipose tissue regulate its mechanical properties, adipogenesis, and lipid droplet growth [37,38]. During obesity, white adipose tissue undergoes expansion through adipocyte hyperplasia and hypertrophy, which are accompanied by continual remodeling of the collagen and ECM. While proper deposition of ECM supports healthy adipose tissue, increased and rigid ECM promotes the local and systemic pathologies associated with obesity [39]. Collagens accumulate around adipocytes, leading to pericellular fibrosis. In gene expression studies of subcutaneous adipose tissue from patients with obesity, fibrosis pathway genes were upregulated [40], and pericellular fibrosis was significantly enhanced [41]. In breast adipose tissue, interstitial collagen around adipocytes was significantly increased in obese patients compared to lean and overweight patients and positively correlated with body mass index (BMI) [42]. Following gastric bypass surgery for weight loss, fibrosis was negatively correlated with fat mass loss [41], suggesting that fat deposition impacts the degree of fibrosis present in adipose tissue. 

Although obesity enhances fibrosis around adipocytes, within mammary tissue, obesity also increases collagen deposition around mammary ducts and preneoplastic lesions [43,44,45]. Interstitial collagen in mammary adipose tissue demonstrated elevated linearity and stiffness in both diet-induced obese mouse models and *ob/ob* mice, which have a mutation in the gene encoding leptin [30]. Further, breast tissue from women with obesity had increased collagen fiber alignment [30]. Expression of various collagen family members, including *Col1a1*, *Col3a1*, and *Col6a1*, was upregulated in the subcutaneous adipose tissue of obese mice [46]. In humans with obesity, *COL5* and *COL6* expression levels were elevated, and elastin levels were reduced in subcutaneous fat [47,48]. In addition to collagen family members, tenascin C and osteopontin are matricellular proteins that are upregulated in obese adipose tissue. Tenascin C expression was enhanced in subcutaneous adipose tissue of obese mice [49], and fibrosis was reduced in mice that the lack expression of tenascin C [50]. Osteopontin was upregulated 40 to 80-fold in subcutaneous adipose tissue of obese mice and was highly expressed by adipose tissue macrophages [51]. Fibronectin was also significantly increased in mammary adipose tissue of obese mice, as well as in breast tumors of obese patients [30]. Thus, obesity leads to increased collagen deposition and changes the characteristics of the ECM in the breast microenvironment.

Enhanced adipose tissue fibrosis in the context of obesity is also associated with a significant increase in tissue stiffness [52]. Cu-dependent lysyl oxidase (LOX), one of five members of the LOX family, contributes to the functions of the ECM by promoting the formation of intra- and intermolecular cross-linkages between ECM components. Elevated levels of LOX were associated with enhanced ECM stiffness [53]. LOX expression was increased in obese subcutaneous adipose tissue of mice and rats [46,54,55,56], and LOX expression in subcutaneous adipose tissue of human subjects positively correlated with BMI [56]. In the mammary fat pad of mice, injection of fibroblasts overexpressing LOX increased tissue stiffness, collagen deposition, and the linearity of collagen fibers [57]. In contrast, when LOX was inhibited, collagen deposition was decreased, and collagen fibers were less linear [57]. Interestingly, LOX expression did not significantly decrease in the adipose tissue of humans nine months after bariatric surgery [56], which may suggest that pathological alterations in fibrosis due to obesity may not be easily reversed following weight reduction.

In addition to its structural role, the ECM also serves as a reservoir for soluble factors, including chemokines. Chemokine binding to resident matrix glycosaminoglycans is important for controlling local concentrations of these soluble factors and their resistance to proteolytic activity and signaling capabilities [58,59]. An increasing number of growth factors, including transforming growth factor beta (TGFβ), insulin-like growth factor, fibroblast growth factor, and hepatocyte growth factor, have been found to associate with ECM proteins or with heparan sulfate [60]. Changes in the composition of the ECM alter the ability of the tissue to bind and retain secreted molecules. For example, TGFβ1 expression is elevated in the adipose tissue of obese mice and humans [61,62,63]. TGFβ1 is produced in a latent form that must undergo extracellular activation prior to receptor binding [64]. Within the ECM, latent TGFβ1 complexes both with latent TGFβ1 binding proteins and matrix components, including decorin, which sequester inactive TGFβ1 until activated [65]. In the obese mammary gland, decorin is enhanced within the ECM and complexes with latent TGFβ1, resulting in increased TGFβ1 storage within the ECM [66]. As TGFβ1 has been implicated in promoting epithelial-to-mesenchymal transition (EMT) in tumor cells and the growth of CAF within the tumor microenvironment [67,68], elevated TGFβ stores within the ECM may impact both the proliferating tumor cells and developing CAF populations.

Fibrosis observed in obese adipose tissue is thought to be in part a result of hypoxia [39], as expanding adipocytes no longer have sufficient blood supply to oxygenate the tissue. In both diet-induced and genetic mouse models of obesity, obese mice had significantly increased hypoxia-induced factor-1 alpha (HIF-1α) expression in adipose tissue [54,69,70]. Concurrent with increased DNA binding of HIF-1α, obese mice demonstrated increased collagen deposition around adipocytes, while treatment of obese mice with β-aminopropionitrile, a LOX family inhibitor, significantly reduced adipose tissue fibrosis [54]. Similarly, collagen deposition around adipocytes and LOX expression was decreased in adipose tissue when HIF-1α was inhibited in obese mice using targeted inhibitors or overexpression of a dominant negative HIF-1α mutant in adipose tissue [70]. As elevated levels of HIF-1α in breast tumors have been associated with metabolic changes in tumor cells, metastasis, and chemotherapy resistance [71], HIF-1α upregulation within the mammary microenvironment prior to tumor formation may contribute to the growth of more aggressive tumors (Figure 1). As in obese adipose tissue, tumor vasculature is significantly more permeable than that of normal tissue [72], which is a characteristic that promotes both immune cell extravasation and fibrosis. 

**Figure 2:**
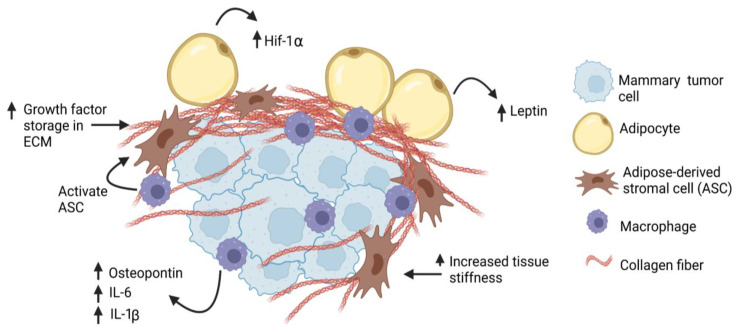
Obesity increases desmoplasia in mammary tumor microenvironment. Tumor-associated macrophages may activate adipose-derived stromal cells (ASC), which may then contribute to the CAF population. Through production of thicker and stiffer extracellular matrix (ECM), breast cancer cells may have increased (↑) exposure to growth factors, hypoxia (Hif-1α), and cytokines, leading to increased proliferation and invasion.

Excess collagen within the mammary gland may enhance cancer risk through the promotion of an aggressive phenotype in premalignant cells. In vitro, MCF10A cells, which model normal breast epithelium, and premalignant MCF10AT cells were more proliferative and invasive when grown on stiff ECM [30,73]. In vivo, injection of MCF10AT cells into mammary fat pads conditioned with LOX-overexpressing fibroblasts led to increased tumor growth and invasion [57]. Using the expression of MMTV-PyMT in a transgenic mouse model of breast density, increased density of mammary collagen enhanced the growth of invasive, rapidly metastatic tumors [73]. Once preneoplastic lesions form, increased collagen deposition and tissue stiffness may also enhance cancer stem-like cells [74,75], which have been implicated in treatment resistance, disease recurrence, and metastasis [76]. Consistent with this, the transplantation of ERα^+^ mammary tumor cells into collagen-dense mammary glands led to increased cancer stem-like cells, circulating tumor cells, and metastasis [77,78]. Elevated collagen deposition and tissue stiffness can also cause aberrant function of endothelial cells, leading to disrupted barrier function and elevated permeability [79]. These defects in endothelial cell function can enhance the release of tumor cells into the circulation and promote metastasis. Together, these results suggest that increased collagen deposition in breast tissue may be implicated in the early promotion of the growth of obesity-associated tumors (Figure 2).

## 3. Obesity and Stromal Cell Function

Although mature adipocytes secrete collagen I, IV, and VI [48,80], significantly greater expression of collagen family members and LOX was observed in cells from the stromal vascular fraction of dissociated adipose tissue compared to mature adipocytes [41,56]. The stromal vascular fraction is a heterogeneous population of cells containing mesenchymal cell types and immune cells and is frequently cultured for in vitro studies. Adherent cells grown from the stromal vascular fraction are referred to as adipose-derived stromal cells (ASC). The ASC are still a heterogenous population that contains mature adipocytes that have lost their lipid droplets, fibroblasts, and adipose stem cells, which have the ability to differentiate into a variety of different cell lineages, including adipocytes, osteoblasts, and chondrocytes, in response to culture stimuli [81]. Adipose stem cells can be enriched using FACS based on cell surface markers CD90, CD105, CD73, and CD44 [82]. Adipose stem cells have been shown to incorporate into the mammary tumor microenvironment to promote cancer growth [83], suggesting that increased numbers of these cells within obese adipose tissue could alter the composition of the tumor stroma.

ASC contributes to ECM deposition and remodeling and has a variety of other roles, including the secretion of growth factors [84,85]. Distinct populations within the ASC of normal breast tissue were identified using gene expression analysis, including one population that had a gene signature similar to tumor stroma [86], presenting the possibility that this population may be primed to become CAF. Within breast tissue, these populations of ASC have distinct effects on the differentiation of breast epithelial cells toward luminal or myoepithelial characteristics during ductal development [87], indicating that obesity-induced changes in ASC also impact epithelial cell populations within phenotypically normal breast tissue. When co-cultured with premalignant MCF10AT1 cells, ASC from obese mice promoted collective migration and enhanced invasion of the MCF10AT cells more effectively than ASC from lean mice [88]. Obesity-induced ASC populations could have direct effects on the growth and behavior of epithelial cells within the mammary gland, which could influence the risk of different subtypes of breast cancer. 

ASC isolated from the mammary glands of obese mice had a decreased potential for differentiation in culture to mature adipocytes and osteocytes than ASC isolated from lean mice [89]. Further, ASC from obese mice demonstrated greater contractile ability [44,89] and higher proliferation rates [30,89]. ASC isolated from obese mice also secreted a stiffer, thicker ECM in vitro, including increased collagen and fibronectin deposition [30]. These studies overall indicate that ASC from the mammary glands of obese mice have a more fibrotic profile than those isolated from lean mice. When cultured on ECM deposited by ASC from obese mice, ASC isolated from lean mice had significantly increased expression of the myofibroblast marker, alpha-smooth muscle actin (SMA) [30]. This myofibroblast phenotype may be dependent upon the increased stiffness of the ECM produced by ASC from obese mice. Enhanced SMA expression was observed in vivo in ASC isolated from the mammary glands of obese mice and breast tissue from women with obesity [30,44,89]. ASC isolated from the mammary glands of obese mice were also found to secrete more TGFβ than their lean counterparts [44], consistent with a more fibrotic phenotype. An increase in the fibrotic profile of ASC promoted by exposure to the obese microenvironment could contribute to the elevated desmoplasia observed in breast tumors from obese patients [30].

In obesity, adipocytes significantly increase expression of leptin, which has been associated with aggressive characteristics in breast tumors [90], and downregulate adiponectin, which has suppressive effects on the growth of breast cancer cells [91,92,93]. In addition to adipokines, adipose tissue secretes multiple inflammatory cytokines, which are produced by both adipocytes and ASC [94]. Gene expression analysis demonstrates that ASC from obese individuals have an upregulation of multiple inflammatory genes, including IL-6, IL-8, and C-C motif chemokine ligand 2 (CCL2) [95,96,97]. Adipocytes differentiated from obese ASC also secreted more IL-6 and leptin than adipocytes from control ASC [98]. In vitro secretion by ASC is altered by other cytokines in the microenvironment. Tumor necrosis factor alpha (TNFα) stimulated the secretion of IL-6, IL-8, and CCL2 from ASC [99], while lipopolysaccharide promoted ASC to express IL-6, IL-8, and TNFα [100]. These results suggest that inflammatory secretion from macrophages recruited into obese adipose tissue may further enhance inflammatory signaling in ASC. In turn, ASC from obese subjects enhanced the polarization of macrophages to an inflammatory phenotype [101]. ASC have been characterized as having a hypoimmunogenic phenotype since they lack the major MHC class II molecules and express only low levels of MHC class I, thus allowing them to evade immune recognition [102,103]. However, in the context of obesity, the immunomodulatory functions of ASC are disrupted [104]. These obesity-altered functions of ASC are similar to CAF within the tumor microenvironment. CAF present during tumor progression also has various immunomodulatory functions [105] and expresses inflammatory cytokines, similar to ASC, which correlate with tumor invasiveness [106].

The appearance of CAF may precede the conversion to malignancy, and CAF are observed surrounding premalignant lesions [107,108]. Distinct CAF populations have been identified that correspond with breast tumor subtypes [105,109]. CAF subpopulations may indicate different cells of origin, including activation of ASC, local fibroblasts, and mesenchymal stem cells [85,110,111,112,113]. A recent single-cell RNAseq study of CAF isolated from tumors that developed in MMTV-PyMT transgenic mice has identified three distinct subpopulations of CAF [114], and further studies are necessary to identify how ASC and adipocytes relate to the normal fibroblast signature identified. In vitro breast cancer cells stimulated mature adipocytes to lose lipid and gain characteristics of fibroblasts, including expression of collagen 1 and fibroblast specific protein-1 (FSP-1), and become more migratory and invasive [115], suggesting that mature adipocytes may also become CAF. Following exposure to breast tumor cell-secreted factors, ASC from obese patients expressed higher levels of CAF markers, including neuron-glial antigen 2, SMA, vascular endothelial growth factor, fibroblast activation protein, and FSP-1, compared to those from lean patients [116]. CAF derived from ASC from the mammary glands of obese mice may also promote the growth of aggressive tumors. In vitro coculture of MCF10DCIS cells with ASC isolated from obese mice led to increased tumorsphere formation and enhanced invasion when cells were cultured on both 2D and 3D substrates [44]. ASC isolated from the mammary glands of obese mice also promoted rapid tumor growth and tumor cell invasion, both in vitro and in vivo [89]. ASC from obese patients enhanced the growth of ERα^+^ tumor cells through a leptin-mediated mechanism [117]. Together, these studies suggest that obesity-induced changes in the mammary microenvironment prior to tumor formation promote populations of CAF conducive to tumor growth and progression.

## 4. Myeloid Lineage Cells and Collagen Deposition in Obesity

Adipose tissue macrophages are central players in obesity-associated inflammation and metabolic diseases [118]. With increasing obesity, macrophages make up 40–50% of the cell population within the adipose tissue of mice, compared to 5% of the cell population in lean mice [119]. Bone marrow transplantation studies revealed that a majority of F4/80^+^ macrophages recruited to the adipose tissue of obese mice were derived from bone marrow [119], and the CCL2-CCR2 signaling axis is a key driver of monocyte recruitment. Obese mice have significantly higher CCL2 expression in adipose tissue compared to lean mice [120,121], including in the mammary gland [122]. While CCL2 expression can be induced by a variety of inflammatory factors [123,124], elevated levels of insulin [121] or TNFα expression [125] may lead to increased CCL2 expression within obese adipose tissue. Macrophage recruitment into obese adipose tissue is reduced in CCL2-deficient mice as well as mice that lack CCR2 [126,127]. The lack of macrophage recruitment of obese adipose tissue in CCR2-null mice is thought to be due to the sequestration of inflammatory monocytes in the bone marrow [128].

Recruited macrophages promote chronic low-grade inflammation in obese adipose tissue [120,129]. Macrophages have been categorized based on the secretion of different types of cytokines as proinflammatory (M1) or anti-inflammatory (M2) activated, although this polarization paradigm fails to capture the complexity of macrophage activation in vivo [130]. In obese adipose tissue, macrophages have been identified as polarized towards an M1 phenotype [131,132], mixed M1 and M2 phenotypes [122,133,134], or M2 polarized, with a gene expression profile similar to tumor-associated macrophages [42]. Histologically, recruited macrophages have been observed to form crown-like structures (CLS) around necrotic adipocytes [135,136]. These CD9^+^ macrophages phagocytose the dying adipocytes and store lipid within obese adipose tissue, leading to their designation as lipid-associated macrophages [137,138]. Exposure to elevated levels of fatty acids and glucose may alter the function of these macrophages to a unique “metabolically activated” phenotype in the obese adipose tissue of both mice and humans [139], and further work has shown that these macrophages cannot be accurately classified as M1 or M2 activated [138,139,140]. In addition to inflammatory cytokines, macrophages within CLS may also locally enhance estrogen concentrations through elevated expression of the enzyme aromatase [141,142]. Elevated estrogen concentrations could have a direct impact on the growth of ERα^+^ breast tumors.

Macrophages have been implicated as critical regulators of fibrosis through the secretion of cytokines and growth factors, which impact other stromal cells in the obese microenvironment. Several studies have identified macrophages as an important source of TGFβ1 [143], and TGFβ1 is a critical regulator of fibrosis in many tissues and organs. Macrophages interact with preadipocytes within the stem cell niche of adipose tissue and impair the ability of preadipocytes to differentiate into adipocytes in obesity [144]. In vitro, co-culture of macrophages with preadipocytes promoted a fibrotic gene signature in the preadipocytes through macrophage secretion of TGFβ family member inhibin βA [145]. These results suggest that macrophages may be important for promoting fibrotic changes in the ASC population. Further, phagocytosis of dying adipocytes within the CLS may enhance fibrosis, as ingesting dead cells has been shown to increase macrophage secretion of TGFβ1 [146,147]. Within CLS, macrophages express macrophage-inducible C-type lectin (Mincle), which is crucial for the expression of fibrosis-related genes [148]. Macrophages may also secrete platelet derived growth factor (PDGF), which stimulates the proliferation, survival, and migration of myofibroblasts [149], as well as IL-1β, which is important for fibrosis within the lung [150,151]. Through the secretion of osteopontin, macrophages also recruit PDGFR^+^ progenitor cells to the sites of adipocyte clearance in CLS [152]. In addition to interactions with other stromal cells, macrophages play an important role in the remodeling of ECM through the secretion of proteases, such as MMP family members, and components of ECM [153]. In MMTV-CCL2 mice, which overexpress CCL2 in mammary epithelial cells, macrophage recruitment and collagen deposition were enhanced around the mammary ducts, and LOX expression was increased in the mammary gland [154], suggesting that these recruited macrophages also enhance fibrosis surrounding the epithelial cells within mammary tissue (Figure 1).

Single-cell RNA-sequencing studies have revealed the complexity of macrophage populations within adipose tissue [155,156]. Different populations of macrophages may have disparate effects on fibrosis within the mammary gland. In particular, there may be differences between resident tissue macrophages, which are derived from the fetal yolk sac and liver [157], and bone marrow-derived macrophages that enhance fibrosis within adipose tissue. Under homeostatic conditions, depletion of resident macrophages in adult mice led to significant increases in collagen deposition within mammary tissue [158], demonstrating the importance of resident macrophages in maintaining collagen deposition and organization. Under conditions of obesity, resident macrophages may play a similar role. Depletion of CD169^+^ resident macrophages exacerbated adipose tissue fibrosis in the visceral fat of obese mice [159]. Recent work has shown that CX3CR1^hi^ macrophages restrain senescence in ASC, which enhances the ability of adipose tissue to expand in response to overnutrition [160]. Together, these results suggest that a population of resident macrophages may in part counteract fibrosis induced by obesity within the mammary gland. In contrast, in a mouse model of renal fibrosis, bone marrow-derived macrophages were shown to differentiate into SMA and collagen 1 expressing myofibroblasts [161,162,163], suggesting that a subpopulation of macrophages may directly enhance fibrosis.

Within the tumor microenvironment, tumor-associated macrophages play critical roles in remodeling the ECM. Reduction of macrophages through anti-CSF1 antibodies in the MMTV-PyMT mouse model led to reduced macrophages, diminished collagen deposition, reduced LOX expression and collagen crosslinks, and decreased lung metastasis, particularly when treatment occurred before the onset of tumor invasion [164]. In mice transplanted with EO771 tumors, clodronate liposome treatment reduced tumor volume and lung metastasis, as well as altered fibrillar collagen microstructure [165]. Similarly, conditional depletion of macrophages during early tumor progression in mice transplanted with CCL2-expressing stromal cells to model the obese microenvironment resulted in lasting reductions in collagen deposition within the growing tumors [166]. In human breast tumors, CD68^+^ and CD163^+^ macrophages were significantly increased at the invasive front compared to the tumor core, and macrophages at the invasive tumor front positively correlated with tissue stiffness [167]. Although obesity enhances desmoplasia in both human breast tumors and mouse models [30,168], it is currently unknown if obesity modifies macrophage function within the tumor microenvironment or if the tumor microenvironment outweighs the condition of obesity in directing macrophage function (Figure 2). Further studies are needed to understand macrophage function within the tumor microenvironment in obesity, as targeted therapeutics are currently in clinical trials [169,170].

In response to the chronic inflammation of obesity, myeloid progenitor cells increase within the bone marrow of both humans and mice [171]. Mast cells are another population of myeloid lineage cells that are recruited to obese adipose tissue [172,173,174,175] and have been implicated in other inflammatory and fibrotic conditions [176,177]. During mammary gland development, mast cells are important for ductal branching, although these cells did not appear to have a significant impact on collagen remodeling in this context [178]. In obese adipose tissue, mast cell secretion of mast cell protease-6 induced expression of collagen V in adipocytes, which reduced the ability of adipose stem cells to differentiate into adipocytes [179]. Further, stabilization of mast cell degranulation in obese mice resulted in reduced expression of TGFβ1 and fibrosis-related genes in adipose tissue [180]. These data suggest that mast cells may play a role in increased adipose tissue fibrosis in obese patients. Mast cells also have an inflammatory phenotype in obesity, characterized by increased expression of IL-6 and CCL2, as well as tryptase and chymase, which are components of mast cell granules [172]. Mast cells may enhance the activity of macrophages in obese adipose tissue for both inflammation and fibrosis by amplifying the secretion of cytokines and growth factors, and additional work is necessary to identify how mast cells impact the mammary gland and tumor microenvironment in obesity.

Fibrocytes are another myeloid lineage cell type that actively participates in fibrosis in wound healing, autoimmune and cardiovascular diseases, and asthma [181]. Since obesity is characterized by both inflammation and fibrosis, fibrocytes may play a role in adipose tissue fibrosis. Fibrocytes originate in the myeloid progenitor cell population in bone marrow and then enter circulation for recruitment to tissue [182,183,184,185,186]. Consistent with their bone marrow origin, fibrocytes express several hematopoietic cell markers, including CD45, CD11b, and CD14, in addition to collagens and glycosaminoglycans, features that are characteristic of fibroblasts [182,187]. Clinically, circulating fibrocytes have been suggested as a prognostic marker of fibrotic disease progression [182,183,184,185,186]. Fibrocytes were recruited into tissue in response to PDGF family members and were inhibited by PDGFR antagonists as well as anti-PDGFR antibodies [188,189]. CCL2 also plays a role in the recruitment of fibrocytes, as CCR2-null mice demonstrated both decreased collagen deposition and fibrocyte numbers in models of kidney and lung fibrosis [190,191]. Once recruited, fibrocytes differentiate in response to multiple cytokines. Depending on microenvironmental conditions, fibrocytes can be stimulated to differentiate into other mesenchymal cell types, including adipocytes, chondroblasts, and osteocytes [192,193,194,194,195], demonstrating the multipotency of this myeloid cell population. In vitro exposure to TGFβ1 stimulated fibrocytes to express SMA [193,196,197,198,199,200], as well as proliferate [201], produce ECM [197], and have increased contractile ability [196]. Fibrocytes also impact surrounding fibroblasts. Conditioned media collected from fibrocytes stimulated the proliferation of fibroblasts through the secretion of PDGF [189]. Coculture of fibrocytes and fibroblasts led to increased expression of SMA and collagen 1A1 in the fibroblast population, which was decreased in the presence of a TGFβ inhibitor [202,203]. Further studies are necessary to determine the role of fibrocytes in adipose tissue fibrosis and mammary cancer risk.

## 5. Weight Loss and Resolution of Fibrosis

Weight loss is frequently recommended as an intervention for patients with obesity to reduce cancer risk. However, the effects of weight loss on breast cancer risk are not well characterized [204]. Intentional weight loss is difficult for patients and challenging to maintain. Bariatric surgery is increasingly common and results in significant, sustained weight loss [205]. Overall, weight loss through bariatric surgery reduces cancer risk relative to obese patients [206,207], including risk for breast cancer [205,208,209]. In one study, bariatric surgery reduced the risk of ERα^−^ breast cancer in premenopausal patients and ERα^+^ breast cancer in postmenopausal patients [210]. However, these weight loss trials are frequently too small to evaluate how weight loss interacts with other factors, such as race, or impact on subsequent breast cancer subtypes, which can all significantly alter breast cancer risk and outcomes [211,212,213]. Some trials also do not follow up with patients for longer than a 6-month period—making it impossible to appropriately explore these parameters [214].

Clinical studies have examined how inflammation in adipose tissue changes over time following weight loss. In the initial 3 months following bariatric surgery, expression of cytokines, including CCL2 and HIF-1α, decreased [215], and CD40^+^ and HAM56^+^ macrophages decreased while CD206^+^ and CD163^+^ macrophages were increased in subcutaneous adipose tissue [216]. These results suggest that macrophage subpopulations may change in the initial period of weight loss. After 12 months, the total number of macrophages and inflammatory CD11c^+^ macrophages were decreased while CD163^+^ macrophages were increased [217,218], which was consistent with changes in macrophage populations in individuals who had lost weight through lifestyle intervention [219,220]. Together, these studies suggest a shift from inflammatory to alternatively activated macrophages following weight loss, which may participate in tissue repair [221]. At a two-year time-point following weight loss, adipose tissue from patients who had maintained weight loss showed an intermediate presence of CD68^+^ macrophages compared to individuals with a BMI in either the lean or obese ranges [222]. However, many of these studies had a small number of patients enrolled as well as limited analysis of changes in adipose tissue. Further work is necessary to identify how weight loss impacts other markers of inflammation within breast tissue. 

Similar to human subjects, weight loss may lead to rapid changes in functionally different macrophage populations within subcutaneous adipose tissue in pre-clinical models. After 2 weeks of weight loss, single-cell RNA-sequencing revealed that macrophages were the largest and most heterogeneous of the leukocyte populations in adipose tissue [156]. During this period, macrophages enriched for lipid metabolism observed in obesity were decreased, while a population of macrophages involved in phagocytosis was increased [156]. These results are consistent with a study that examined adipose tissue 3 days after weight loss; there was an influx of F4/80^+^ macrophages, but not increased numbers of CLS [223]. Enhanced numbers of macrophages early in weight loss may result from proliferation of resident adipose tissue macrophages [224]. Following 5–10 weeks of weight loss, macrophage numbers decreased but did not return to levels observed in lean mice. Weight loss due to calorie restriction or switching to a low-fat diet had similar impact on macrophages resulting in reduced total numbers of macrophages [223,224,225,226] and decreased CLS [225,226,227].

Adipose tissue fibrosis is a potential complicating factor that may lead to reduced weight loss following bariatric surgery. Increased collagen in subcutaneous adipose tissue was negatively correlated with fat mass loss following surgery [41,52,228]. Collagen fibrosis may also be a long-lasting feature within adipose tissue of patients who have lost weight. In subcutaneous adipose tissue of patients who maintained weight loss for at least 2 years, collagen fibrosis remained similar to patients with obesity [222]. After 12 months, weight loss induced by bariatric surgery resulted in increased collagen in adipose tissue of patients, although this may be attributed to elevated collagen fragments rather than enhanced fibrosis [217]. In one study, diet-induced weight loss led to decreased LOX expression in subcutaneous adipose tissue, as well as downregulation of ECM-associated gene pathways [229], while in another study, LOX expression did not decrease in adipose tissue 9.5 months following bariatric surgery [56]. Overall, longer clinical studies, as well as mouse models, are needed to determine how adipose tissue remodeling during weight loss affects the types of collagen being actively synthesized as well as the progression of collagen degradation.

**Figure 3:**
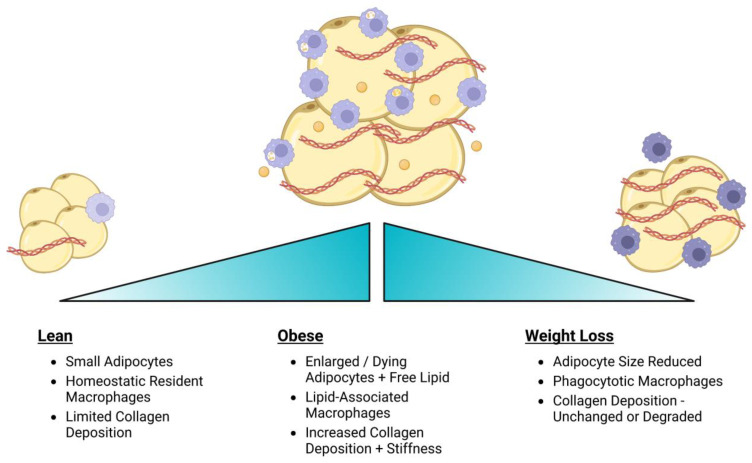
Weight loss modulates the adipose tissue microenvironment. Obesity leads to changes in adipocyte size, macrophage diversity, and collagen deposition, which are only partially reversed with weight loss.

While there are no long-term studies in mice examining the impact of weight loss on adipose tissue fibrosis, the effects of exercise on inflammation and collagen deposition in adipose tissue have been examined. In one study, mice were fed a high-fat diet for 12 weeks followed by a switch to low-fat diet to induce weight loss or continued consumption of high-fat diet with access to a wheel to exercise for 3 weeks. Adipose tissue of weight loss mice demonstrated similar numbers of macrophages compared to obese mice, while the mice that exercised had intermediate macrophage numbers compared to weight loss and obese mice [230], demonstrating that exercise was more effective at reducing adipose tissue inflammation over a short period of weight loss. In another study, mice were fed a high-fat diet for 12 weeks, followed by continuation of the high-fat diet with treadmill exercise for 12 more weeks. Levels of F4/80^+^ macrophages in adipose tissue of mice that exercised were intermediate between lean and obese mice [231], similar to the previous study. Further, while obesity increased collagen deposition in adipose tissue, exercise reduced collagen deposition and gene expression of LOX and other fibrotic genes to levels similar to lean mice [231]. These data suggest that weight loss through exercise may reduce not only inflammation but also fibrosis in adipose tissue (Figure 3).

While weight loss through bariatric surgery has been shown to reduce risk of breast cancer [205,208,209], results in pre-clinical models have been less clear. In obese mice that received vertical sleeve gastrectomy, one of the common methods of bariatric surgery, the surgical intervention was not as effective as weight loss through dietary intervention for reducing mammary tumor growth despite achieving similar levels of weight loss [232]. However, the surgical intervention for weight loss significantly increased expression of PD-L1 within the tumor microenvironment, suggesting that immunotherapy targeting immune checkpoint inhibitors may be more useful for this group of patients [232], although more work is necessary to confirm this finding in breast cancer patients. Obese mice switched from a high-fat diet to a low-fat diet to induce weight loss in tumor cell transplant models and transgenic mouse models resulted in reduced tumor latency or decreased incidence [232,233,234,235]. However, other studies have suggested that weight loss through dietary intervention alone did not completely reverse tumor growth at the rates of control mice [236,237,238]. Mechanistically, weight loss through a reduced calorie dietary intervention may not reverse methylation patterns within the mammary gland [238,239], resulting in a gene expression pattern associated with poor prognosis within the tumor cells of formerly obese mice, similar to obese mice [238]. Together, these studies suggest that weight loss has nuanced effects on mammary tumor growth and progression, and further studies in both pre-clinical models and human subjects are needed to understand how weight loss alters fibrosis during tumor growth and response to therapy.

## 6. Conclusions

As the number of individuals with obesity continues to rise, understanding how obesity increases breast cancer risk and transforms the breast tumor microenvironment is critical for designing interventions and therapeutic strategies. Desmoplasia within breast tumors is associated with more aggressive clinical behavior due to reduced patient relapse-free survival and decreased response to therapy [32,33]. Obesity generates an environment conducive to the formation of desmoplastic tumors and sets the stage for the early progression of tumors, including the invasion of cancer cells into surrounding tissue and metastasis. Prior to tumor formation, obesity enhances collagen and ECM deposition within the mammary gland, surrounding adipocytes and epithelial cells. This increased collagen elevates tissue stiffness, which may enhance invasion of epithelial cells following transformation and contribute to early tumor progression [240]. Obesity also promotes chronic, macrophage-driven inflammation within adipose tissue. Macrophages secrete multiple factors, such as osteopontin, IL-1β, and IL-6, which have been implicated in enhancing adipose tissue fibrosis, although less is known about the impact of obesity on macrophage function within tumors. Further, aberrant cytokine signaling from macrophages leads to an activated phenotype in ASC, similar to that observed in CAF, and promotes the deposition of ECM. Although weight loss is recommended for the resolution of obesity, significant questions remain regarding the ability of weight loss to reverse obesity-induced changes in fibrosis and inflammation within the mammary gland microenvironment. While obesity-induced changes in ASC and macrophages in subcutaneous adipose tissue have been considered, little is known about how obesity alters the complement and function of CAF populations or macrophages within mammary tumors. Additional studies are necessary to understand how changes to the stromal cells of the mammary gland due to obesity shape the microenvironment of developing mammary tumors.
